# The influence of different intervention measures on improving mobile phone addiction among teenagers or young adults: a systematic review and network meta-analysis

**DOI:** 10.3389/fpsyt.2025.1629251

**Published:** 2025-09-17

**Authors:** Ying Li, Zhiyu Zhang, Jianhua Zhang, Qiuyan Zhang, Zhaojun Luo, Dan Tao

**Affiliations:** ^1^ College of Sports Science, Jishou University, Jishou, Hunan, China; ^2^ Faculty of Health Sciences and Sports, Macao Polytechnic University, Macao, Macao SAR, China; ^3^ School of Physical Education and Arts, Hunan University of Medicine, Huaihua, Hunan, China; ^4^ Physical Education Department, Beijing Wuzi University, Beijing, China; ^5^ Football College, Wuhan Sports University, Wuhan, China

**Keywords:** mobile phone addiction, teenagers, young adult, network analysis, meta

## Abstract

**Objective:**

This study employed network meta-analysis to evaluate the impact of several exercise interventions on mobile phone addiction. The aim is to determine the most effective exercise intervention measures and provide a reference for future intervention measures to improve mobile phone addiction.

**Method:**

Systematically search for relevant literatures in domestic and foreign databases such as Web of Science, PubMed, Embase, Cochrane Library, China Knowledge, Wanfang, etc. We evaluated the risk of bias according to the revised Cochrane Randomized Trial Risk Tool and conducted traditional and web-based meta-analyses using Review Manager 5.3 and Stata 14.0.

**Result:**

Traditional Meta-results showed that all interventions were superior to the control group in improving mobile phone addiction (SMD= -1.38, 95%CI=-1.75, -1.01). Network meta-analysis shows that Badminton and Mindfulness-Based Therapy (MBT) have better improvement effects on mobile phone addiction among teenagers than other forms of exercise.

**Conclusion:**

All kinds of interventions have a significant impact on reducing mobile phone addiction. Badminton and MBT have more advantages in improving mobile phone addiction. However, due to the influence of the sample size and the quality of the included literature, it is recommended to further verify the results in the future.

## 1 Introduction

Smartphones have become an inseparable part of daily life. Their use makes human life easier because they have many convenient functions and software applications (1). While mobile phones bring convenience, they also bring potential risks, leading to new behavioral problems, among which is mobile phone addiction (2). Mobile phone addiction is the theoretical criterion for defining behavioral addiction, including psychological (craving, cognitive prominence, loss of control, emotional correction), physical dependence (tolerance and withdrawal symptoms), significance, impulsiveness, spotlight behavior, and relapse (3–5). The core signs and symptoms of mobile phone addiction include obsessive thoughts about the phone (craving), spending extra time on the smartphone (tolerance), and experiencing anxiety when the smartphone is unavailable (withdrawal) (6, 7). Mobile phone addiction reduces activity levels and leads to an increase in fat and a decrease in muscle mass (8). It also lowers the sleep quality of teenagers, causes damage to the lens, and leads to immune system dysfunction (9). In addition, teenagers’ skulls are thinner, and their brain tissue is more electrically conductive. They are more likely to absorb mobile phone radiation than adults and have a higher risk of developing brain tumours than adults (10). In addition, mobile phone addiction can also cause anxiety in various aspects, such as self-existence, social interaction, and academic studies. The cognitive dissonance and negative automated thinking it triggers can further exacerbate depression, leading to more suicidal thoughts among teenagers. Furthermore, mobile phone addiction is negatively correlated with the academic performance of adolescents (11). Excessive use of mobile phones can weaken students’ inhibitory control, working memory and attention, affect teaching coherence and hinder establishing a supportive, cooperative learning environment, and those at high risk also find it more challenging to improve school adaptability through self-regulation (12). Mobile phone addiction has seriously affected the physical fitness and intelligence level of teenagers. Relevant reports indicate that mobile phone addiction has become a label for teenagers. Research shows that the incidence of mobile phone addiction among teenagers is 70% (13–15), and 21.3% of college students in China are addicted to smartphones (16). In Italy, 30% of teenagers are addicted to mobile phones (17). Therefore, it is extremely urgent to solve the problem of teenagers’ addiction to mobile phones.

At present, taking scientific and effective measures to reduce teenagers or young adults mobile phone dependence has become the focus of multidisciplinary attention. The existing intervention measures for mobile phone addiction mainly include group counseling intervention, cognitive behavioral intervention and exercise intervention, etc. At present, there are also many clinical randomized controlled trials (RCTS) to verify the intervention effects of various measures (18, 19), and some scholars have conducted research through traditional meta-analysis (20). However, there is a lack of direct comparisons among the intervention effects of different measures. Network meta-analysis (NMA) is an extension of traditional pairwise meta-analysis, with the advantage of simultaneously comparing the effectiveness of multiple interventions for a specific outcome. Even when direct comparisons between two interventions are not available within the network structure, NMA can still calculate indirect comparisons (21). By integrating both direct and indirect evidence, NMA facilitates the ranking of various interventions’ effectiveness (22). However, research on the efficacy of different interventions for mobile phone addiction remains limited. Therefore, we studied and explored the influence of varying intervention methods on mobile phone addiction, and classified the interventions in detail to determine the best intervention methods for improving mobile phone addiction, guiding teenagers or young adults with mobile phone addiction to choose the best intervention methods, and reducing mobile phone addiction among teenagers or young adults.

## 2 Methods

This study was reported per the PRISMA NMA guidelines (23). The review protocol was registered with the International Prospective Register of Systematic Review (PROSPERO CRD420251127958).

### 2.1 Search strategy

The computer searched PubMed, Web of Science, Embase, Cochrane Library, CNKI, and other databases, and the search period was established until April 28, 2025. The search takes the way of combining subject words and free words. We conducted a search using Pubmed as an example. For the search strategy, please refer to the **Supplementary Materials** (**Appendix 1**).

### 2.2 Study selection

The inclusion criteria for study selection were based on the PICOS methodology (Participants, interventions, comparators, outcomes, and study design) (23), shown in **Table 1**.

**Table 1:** Inclusion and exclusion criteria.

Category	Inclusion criteria	Exclusion criteria
Population	Teenagers or Young adults who have reached the level of mobile phone addiction after assessment	Medically diagnosed severe mental disorders
Interventions	Aerobic Aerobics (AA), Badminton, Baduanjin, Basketball, Biofeedback, Tai Chi (TC), Table tennis (TT), Jump rope (JR), Combined Intervention (CI), Mindfulness-Based Therapy (MBT), Cognitive Therapy (CT), Sanda, Volleyball, Yoga	
Comparisons	Control group (CG)	
Outcomes	Using Smartphone Addiction Scale Shortened Version (SAS-SV), The mobile phone addiction index (MPAI). SAS-SV or MPAI has good reliability and validity (24, 25).	
Study	Randomized controlled trial; published in English or Chinese	duplicate publications; conference papers and review articles.

The types of interventions were based on the exercise interventions actually reported in the included studies.

### 2.2 Data extraction

Following database screening, all identified records were imported into Note Express software for duplicate removal. Two independent investigators systematically applied our pre-defined PICOS criteria through title/abstract screening. Potentially eligible studies were then transferred to Zotero software (v6.0.30) for full-text evaluation and data extraction. Discrepancies were resolved through consultation with a third reviewer. Standardized extraction forms captured (1): basic characteristics (first author, publication year, region) (2); participant demographics (sample size, age range) (3); intervention protocols (modality, weekly frequency, duration); and (4) outcome measures with corresponding assessment methods.

### 2.3 Risk of bias assessment

The risk of bias was assessed independently by two reviewers and by a third reviewer using the tools provided by the Cochrane Collaboration (26), including sequence generation, hidden assignment, blinking, incomplete outcome data, non-selective reporting of results, and other sources of bias. Each criterion was judged to have a low, unclear, or high risk of bias.

### 2.4 Data analysis

Meta-analysis was performed using Rev Man 5.3 software. The standardized mean difference (SMD) with 95% confidence intervals was selected as the effect measure for continuous outcomes, and a random-effects model was applied to pool effect sizes across studies. A 95% confidence interval (95% CI) represented each effect size. *I^2^* was used to determine the heterogeneity of effect indicators among different studies quantitatively. An *I^2^* >50% or ap value of 0.10 or less for the Q test was interpreted as indicating substantial heterogeneity (26). When the heterogeneity was significant, the random effects model was used; otherwise, the fixed effects model was used. Subgroup analysis was based on movement characteristics and population characteristics. The source of heterogeneity was explored through sensitivity analysis for the studies with significant heterogeneity. Sensitivity analysis was used to test whether the source of heterogeneity was due to one of the original studies.

NMA was also conducted to perform a random-effects multivariate NMA for pooled estimates within the frequentist framework (27). We conduct NMA based on the frequency-based framework through Stata16.0 (*network and mvmeta packages*). The standardized mean difference (SMD) and its 95% confidence interval were used as combined statistics, and the direct and indirect comparison evidence was integrated through the multivariate random-effects model. The geometry of the network is summarized into a *networkplot*, in which the lines connecting the nodes represent direct head-to-head comparisons between interventions, and the size of each node and the thickness of each line connecting the nodes are directly proportional to the number of studies. Draw the network contribution graph (*netweight package*) and calculate the contributions of each direct comparison. The local-global inconsistency test (*network meta inconsistency/consistency*) is adopted, supplemented by the node splitting method to identify the inconsistency of specific loops. The surface under the cumulative ranking curve (SUCRA) was used to rank and compare the effects of the different interventions. SUCRA values range from 0 to 100, where 100 indicates the best treatment with no uncertainty, and 0 indicates the worst treatment without uncertainty (28). Moreover, a network funnel plot was generated to check for publication bias (*netfunnel*).

## 3 Results

### 3.1 Study selection

**Figure 1:**
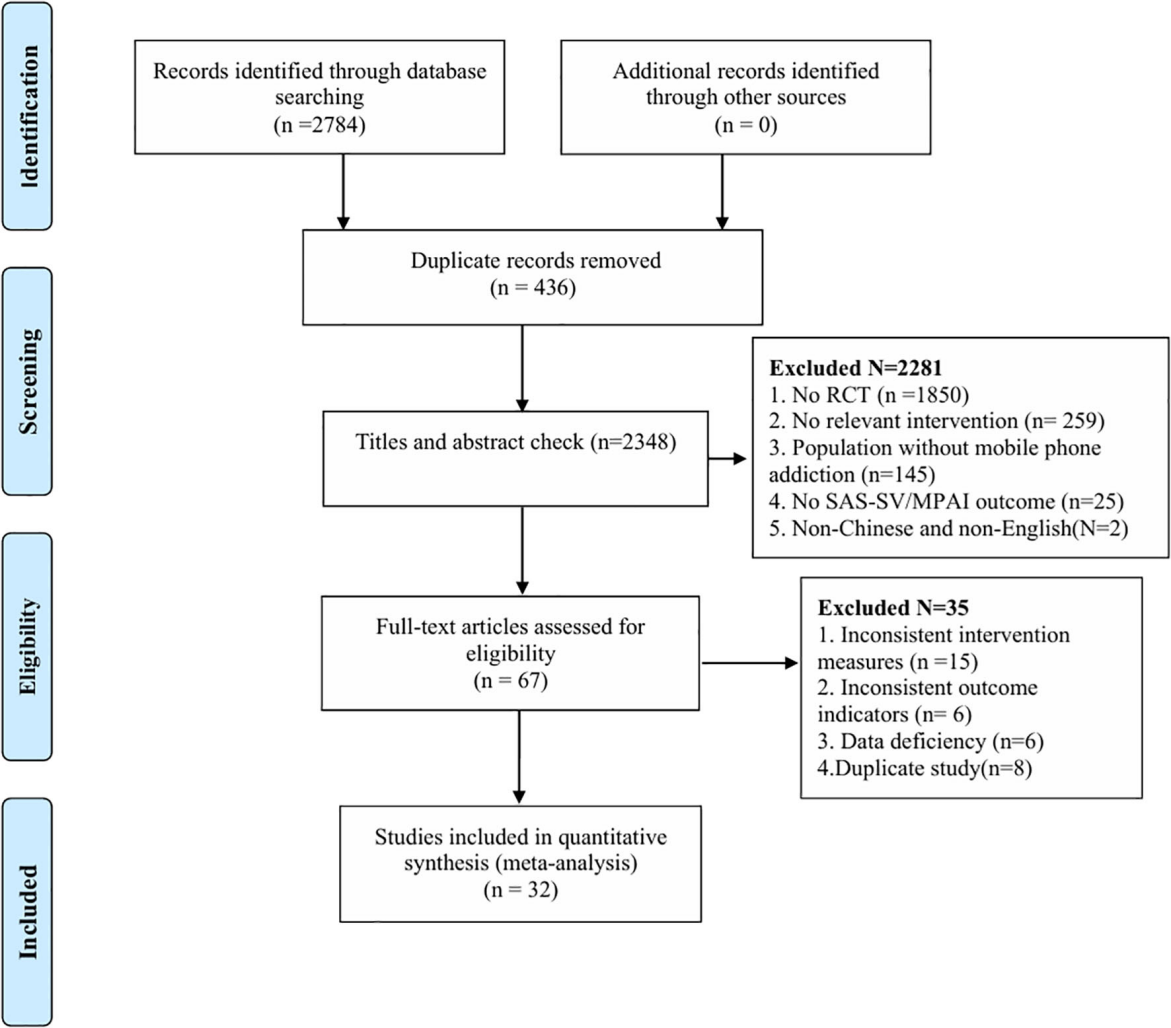
PRISM flow diagram.

Our research obtained a total of 2,784 records. After deleting 436 duplicate records, the titles and abstracts of 2,348 studies were screened. Subsequently, read the remaining 67 articles in full. After reading the full text, we eliminated 35 articles that did not conform to the research. Ultimately, 32 studies were included in the meta-analysis. The research flow chart is shown in **Figure 1**.

### 3.2 Basic information included in the study

As shown in **Table 2**, a total of 2,891 teenagers or young adults addicted to mobile phones were involved in the 32 included studies. In terms of result measurement, 15 studies used MAPI to assess the severity of mobile phone addiction, 3 studies used SAS-SV for assessment, and 14 studies used MPATS for assessment. 15 studies were published before 2020, and 17 studies were published after 2020.

**Table 2:** Detailed characteristics of the included studies.

Author(s)	Publication year	Country	Study population	Age	Intervention method	Sample	Intervention duration	Intervention frequency	Outcome measures
Liao (29)	2022	China	University	20.12 ± 1.54	Basketball/CG	8/8	6 Weeks	NA	MPAI
Wang (30)	2021	China	Middle School	12-13	Basketball/CG	17/16	9 Weeks	Twice a week	SAS-SV
Yang (31)	2022	China	High school student	16.45 ± 2.02/16.74 ± 1.67/16.28 ± 1.96	AA/Badminton/CG	36/36/36	12 Weeks	Three times a week	MPAI
Zhang (32)	2023	China	University	20.11± 0.64	CI/TC/CG	30/30	8 Weeks	Three times a week	SAS-SV
Zeng (33)	2024	China	University	19.00 ± 0.43/19.00/19.00 ± 0.35	Badminton/CG	17/17/17	10 Weeks	Twice a week	MPATS
Yu (34)	2023	China	University	18.83 ± 0.87/18.87 ± 0.94	AA/CG	30/30	16 Weeks	Three times a week	MPAI
Wu (35)	2022	China	University	NA	TT/CG	10/17/23	12 Weeks	Twice a week	MPATS
Yang (36)	2020	China	University	NA	CI/CG	30/30	8 Weeks	Twice a week	MPATS
Ge (37)	2015	China	University	21.24 ± 1.08	Volleyball/CG	18/18	18 Weeks	Three times a week	MPAI
Li (38)	2020	China	University	21.05 ± 1.02/21.05 ± 1.65	CI/CG	16/16	12 Weeks	Three times a week	MPATS
Zhang (39)	2022	China	University	20.27 ± 1.95	Biofeedback/CI/CG	17/17/20	8 Weeks	Twice a week	MPATS
Xie (40)	2019	China	University	NA	Baduanjin/CG	162/152	8 Weeks	Five times a week	MPAI
Liu (41)	2019	China	University	19.21 ± 1.02/18.95 ± 0.89/18.77 ± 1.29/19.71 ± 1.77	Basketball/Baduanjin/GC/CG	31/31/30/34	12 Weeks	Three times a week	MPAI
Liu (42)	2022	China	University	NA	Basketball/Baduanjin/GC/CG	31/31/30/34	10 Weeks	Twice a week	MPAI
Sheng (43)	2017	China	University	NA	GC/CG	2/6/6	8–10 Weeks	Once a week	MPAI
Niu (44)	2020	China	University	21.33 ± 2.05/22.94 ± 2.13	MBT/CG	400/400	12 Weeks	Twice a week	,MPAI
Li (45)	2019	China	University	20.14 ± 1.33/20.06 ± 1.03	MBT/CG	28/31	8 Weeks	Once a week	MPATS
Shen (46)	2022	China	University	NA	MBT/CG	34/34	8 Weeks	Once a week	MPAI
Du (47)	2024	China	University	20.39 ± 1.20/20.35 ± 1.17	MBT/CG	28/31	8 Weeks	Once a week	MPATS
Dai (48)	2018	China	University	NA	MBT/CT/CG	27/20/20	4 Weeks	Once a week	MPATS
Feng (49)	2015	China	University	NA	GC/CG	10/10	8 Weeks	Once a week	MPATS
Qing (50)	2019	China	University	NA	GC/CG	34/34	8 Weeks	Once a week	MPATS
Zhou (51)	2021	China	University	NA	GC/CG	8/41	8 Weeks	Once a week	MPATS
Deng (52)	2016	China	University	18.4 ± 0.5/18.9 ± 0.2	GC/CG	7/7	4 Weeks	Once a week	MPAI
Zhou (53)	2021	China	University	NA	CT/CG	12/12	8 Weeks	Once a week	MPATS
Tao (54)	2021	China	University	18.95 ± 0.89/19.21 ± 1.02/19.71 ± 1.77	Basketball/Baduanjin/CG	33/33/34	12 Weeks	Three times a week	MPAI
Pal (55)	2022	India	University	20.2 ± 2.2/19.7 ± 1.5	Yoga/CG	142/142	NA	Six times a week	SAS-SV
Haihong (56)	2023	China	University	19.1 ± 0.5	MBT/CG	28/28	8 Weeks	Once a week	MPAI
YUKUN (57)	2018	China	University	21.3 ± 1.3	MBT/CG	41/29	8 Weeks	Once a week	MPATS
Zhou (58)	2022	China	University	18.93 ± 1.90/19.22 ± 0.86	Sanda/CG	121/116	10 Weeks	Once a week	MPATS
Zhang (59)	2020	China	University	20.1/20.5	MBT/CG	17/15	8 Weeks	Once a week	MPAI
An (60)	2020	China	University	NA	GC/CG	8/8	8 Weeks	Once a week	MPATS

### 3.3 Methodological quality assessment

**Figure 2:**
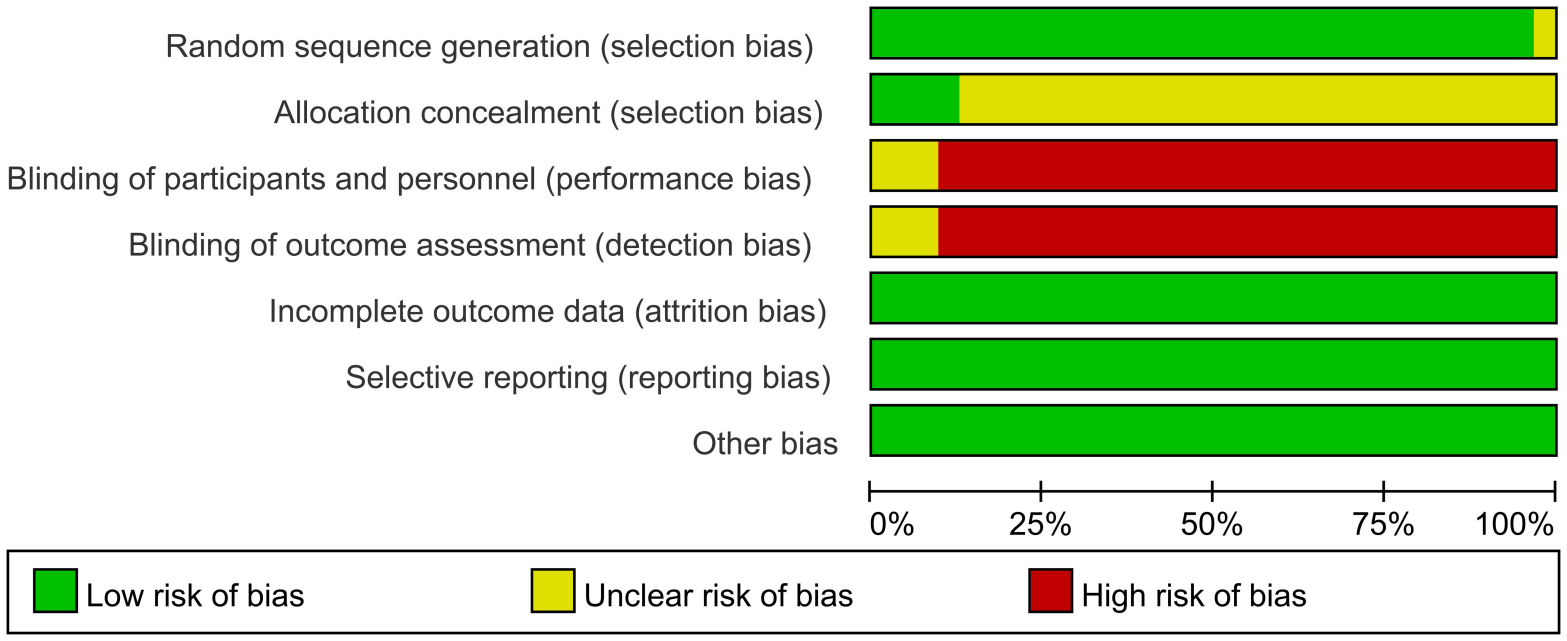
Summary of risk of bias.

The methodological quality of the included 32 articles was evaluated. The summary of the risk of bias assessment is shown in **Figure 2**.

### 3.4 Meta-analysis

**Figure 3:**
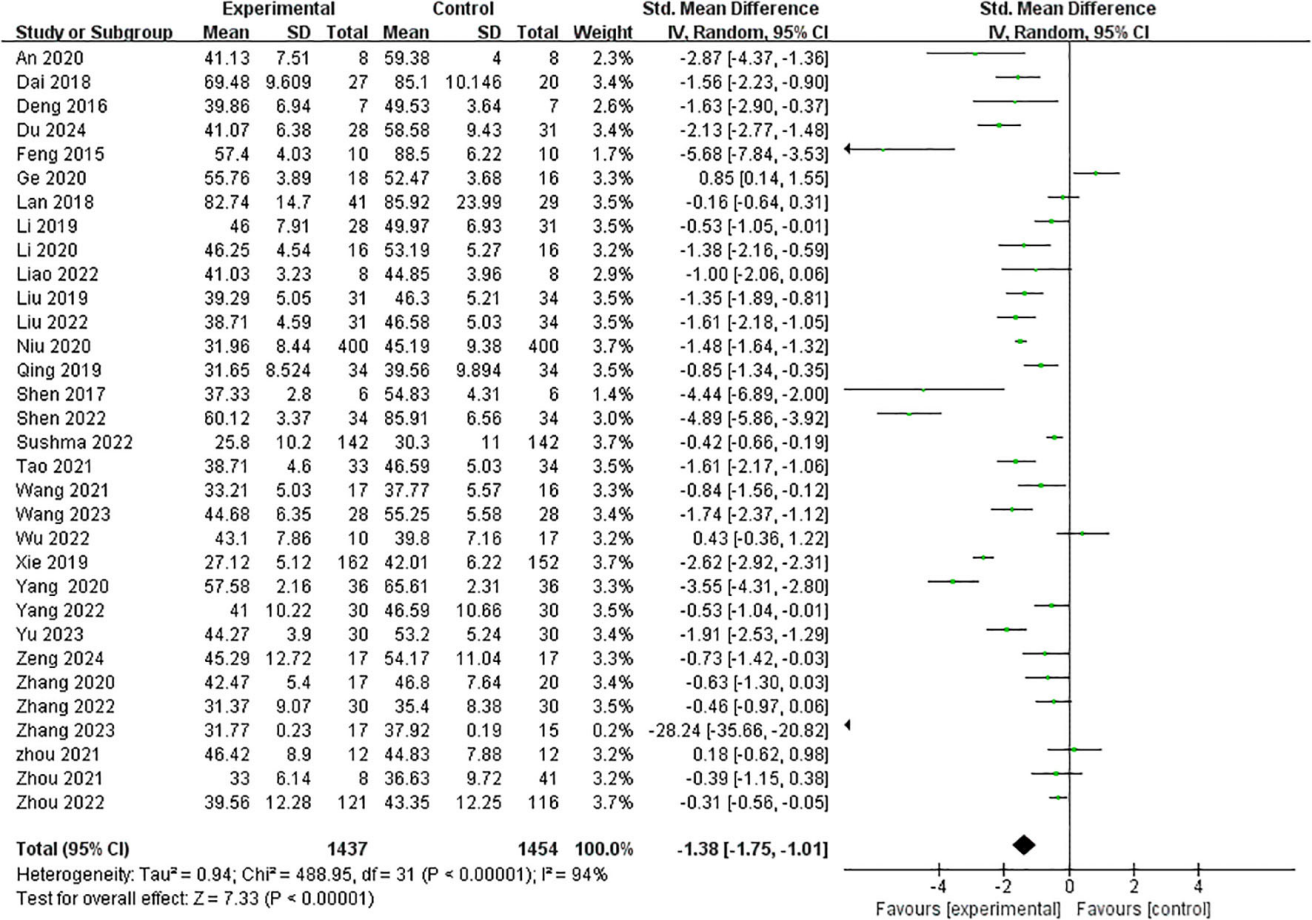
Impact of interventions on Mobile phone addiction.

The effect of the intervention measures was compared with that of the control group. A meta-analysis was conducted on 32 studies. The overall result is shown in **Figure 3**. Compared with the blank control group, exercise intervention had a significant effect on improving mobile phone addiction in adolescents [SMD= -1.38, 95%CI (-1.75, -1.01), p < 0.001], and *I^2^* showed significant heterogeneity (*I^2^* = 94%, p < 0.001).

#### 3.4.1 Subgroup analysis

We conducted subgroup analyses based on sample size, Intervention mode, outcome measurement, Intervention duration, Year of publication, and intervention frequency. There was no statistically significant difference between the two groups in terms of publication year and Intervention frequency, sample size and Intervention duration (P > 0.05). In terms of the outcome measurement, and Intervention mode, the differences between subgroups were statistically significant (p < 0.05), as shown in **Table 3**.

**Table 3:** Subgroup analysis to assess the effect of interventions on adolescents’ intervention addiction.

Variable	Number of trials	Sample size		Meta-analysis			Heterogeneity		
		Experimental	Control	SMD	CI	P^a^	I^2^	Chi^2^	P^b^
All	32	1437	1454	-1.38	-1.75, -1.01	—	94%	488.95	<0.001
Year of publication									
Before 2020	15	835	808	-1.58	-2.17, -1.00	0.38	94%	229.76	<0.001
After 2020	17	602	646	-1.25	-1.73, -0.76		93%	214.75	<0.001
Sample size									
≤50	15	198	229	-1.41	-2.17, -0.66	0.87	90%	133.88	<0.001
>50	17	1239	1225	-1.48	-1.93, -1.04		95%	339.17	<0.001
Outcome measurement									
MPAI	15	853	846	-1.97	-2.58, -1.37	<0.001	94%	236.24	<0.001
SAS-SV	3	189	188	-4.43	-6.29, -2.58		0	0.04	0.56
MPATS	14	395	420	-0.91	-1.34, -0.53		84%	79.33	<0.001
Intervention duration									
4–8 Weeks	21	952	962	-1.60	-2.08, -1.11	0.14	93%	289.33	<0.001
12–16 Weeks	11	485	492	-1.05	-1.60, -0.50		83%	138.83	<0.001
Intervention mode									
Exercise Intervention	17	749	748	-1.04	-1.53, -0.55	0.03	94%	272.90	<0.001
Psychological Intervention	15	688	706	-1.91	-2.54, -1.28		93%	194.85	<0.001
Intervention frequency									
Once a week	15	409	422	-1.91	-2.62, -1.19	0.14	94%	216.48	<0.001
2–3 times a week	14	716	730	-1.06	-1.50, -0.63		90%	126.40	<0.001
Other	3	312	302	-1.36	-3.06, 0.35		98%	126.09	<0.001

#### 3.4.2 Sensitivity analysis

Sensitivity analysis of the included literature showed that no single study changed the overall outcome.

#### 3.4.3 Publication bias

**Figure 4:**
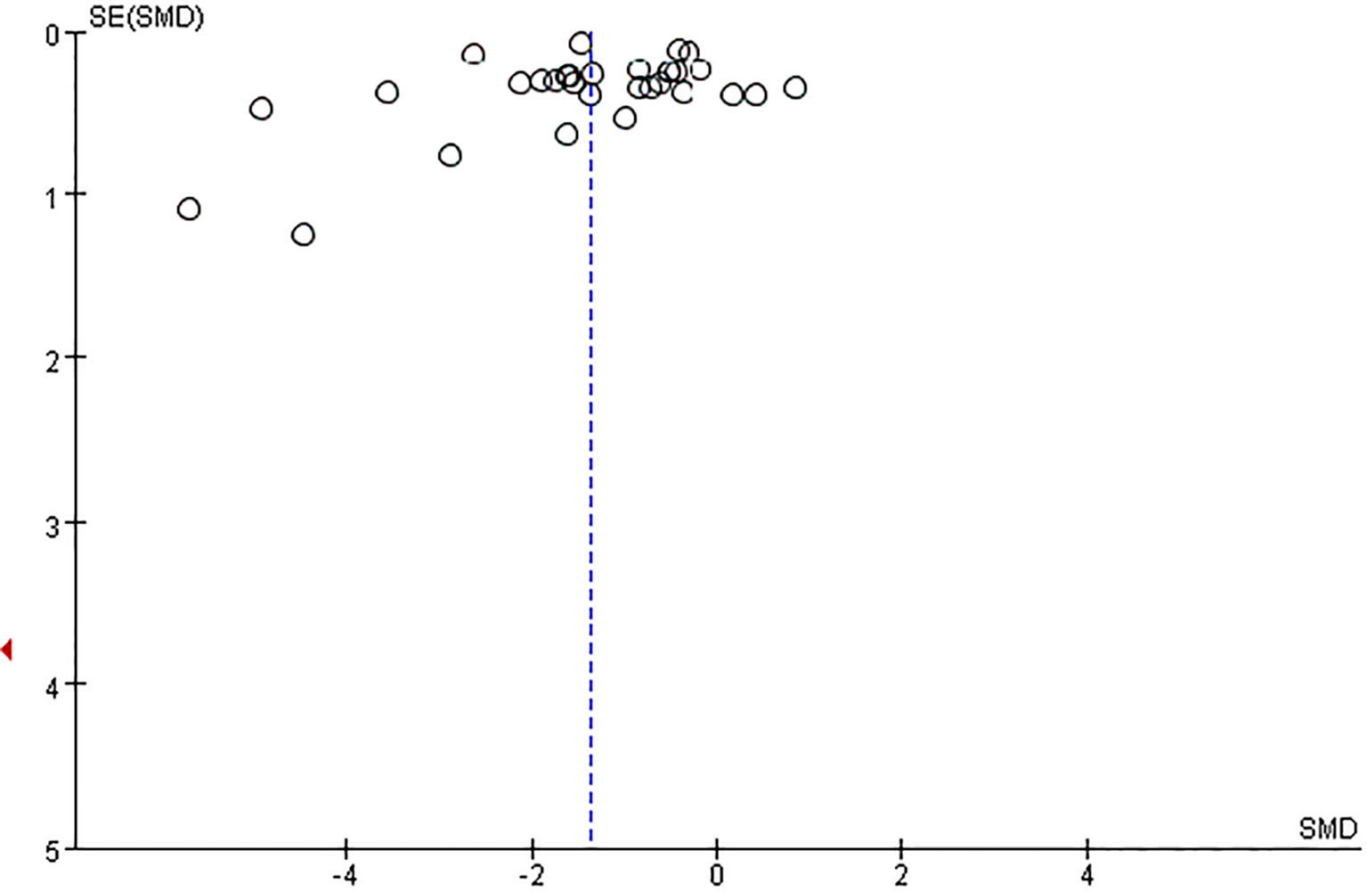
Funnel plot for the publication bias of adolescents’ Mobile phone addiction.

The funnel plot showed potential publication bias (**Figure 4**). We further conducted the test through Begg, and the result of the Egger’s test was P = 0.450 (P > 0.05), indicating that the probability of surface bias in the included studies was relatively low.

### 3.5 Network meta-analysis

To examine the differences in effects among the different interventions, network meta-analyses were further performed.

#### 3.5.1 Network diagram

**Figure 5:**
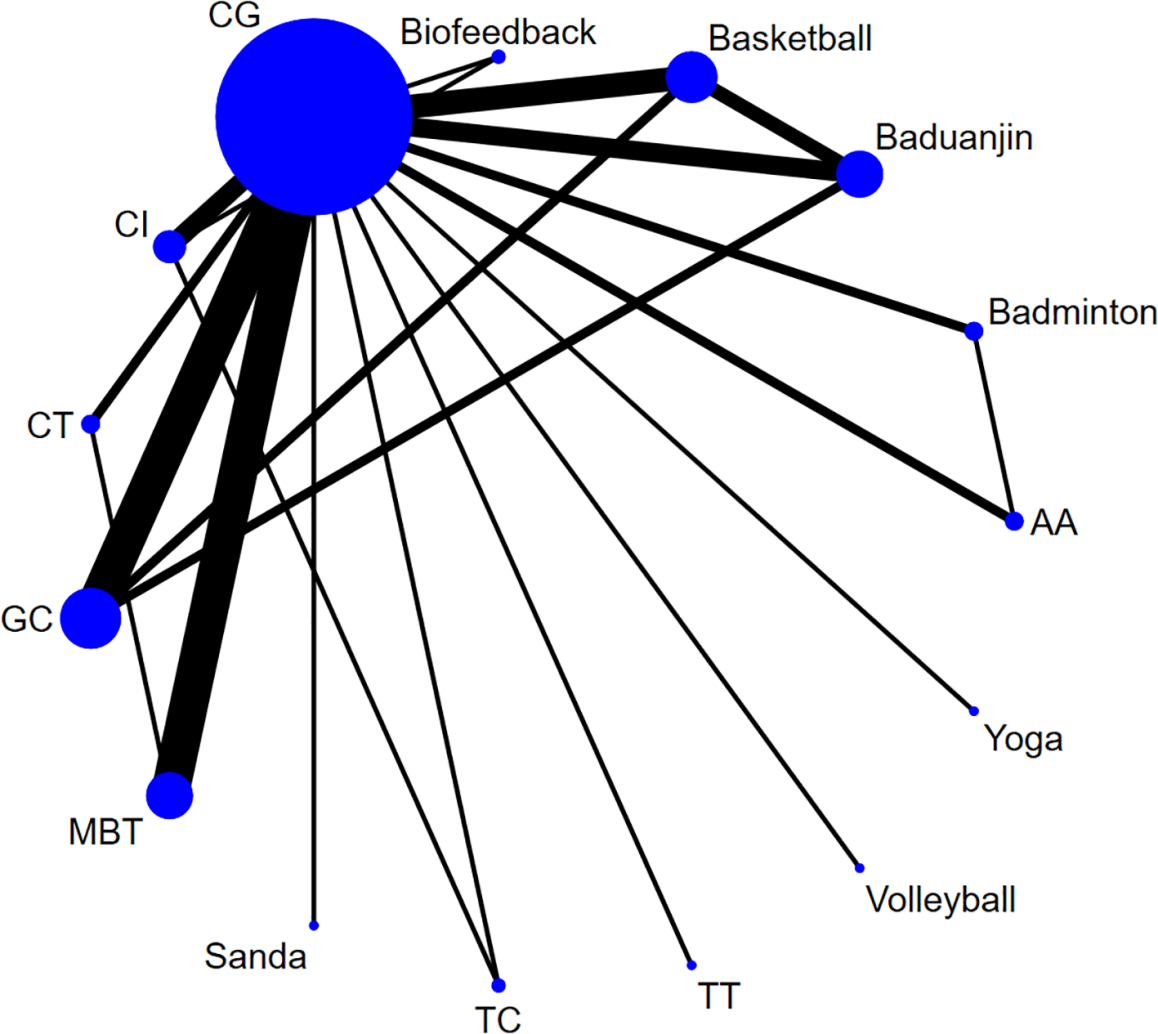
Network diagram of mobile phone addiction. Aerobic Aerobics (AA), Tai Chi (TC), Table tennis (TT), Jump rope (JR), Control group (CG), Combined Intervention (CI), Mindfulness-Based Therapy (MBT), Cognitive Therapy (CT), Group Counseling (GC).

As shown in **Figure 5**, the dots in the figure represent the number of subjects in each group; the larger the dots are, the larger the sample size of the subjects. The lines connecting the dots represent the number of original studies directly compared in pairs; the thicker the lines are, the more original studies there are.

#### 3.5.2 Inconsistency of the network

The inconsistent model was adopted for verification. The result (P=0.991) indicated that the model inconsistency was not significant. The inconsistency and local inconsistency tests were conducted again. We did not find significant inconsistencies in all the results.

#### 3.5.3 Results of network meta-analysis

**Figure 6:**
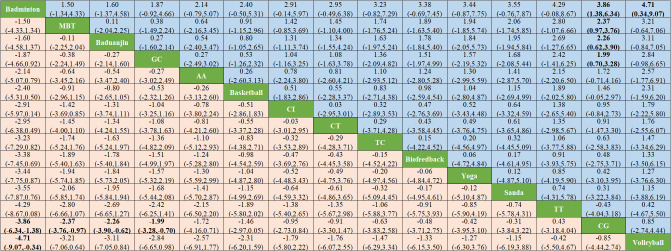
Results of network meta-analysis.

Network meta-analysis shows that badminton is significantly superior to CG (SMD -3.86, 95% -6.34 to -1.38) and volleyball (SMD -4.71, 95% -9.07 to -0.34). MBI was significantly superior to CG (SMD -2.37, -3.76 to -0.97), as shown in **Figure 6**. The forest plots of the comparisons that meet the conditions are shown in **Supplementary Materials** -**Appendix 2**.

#### 3.5.5 Intervention effect ranking

**Figure 7:**
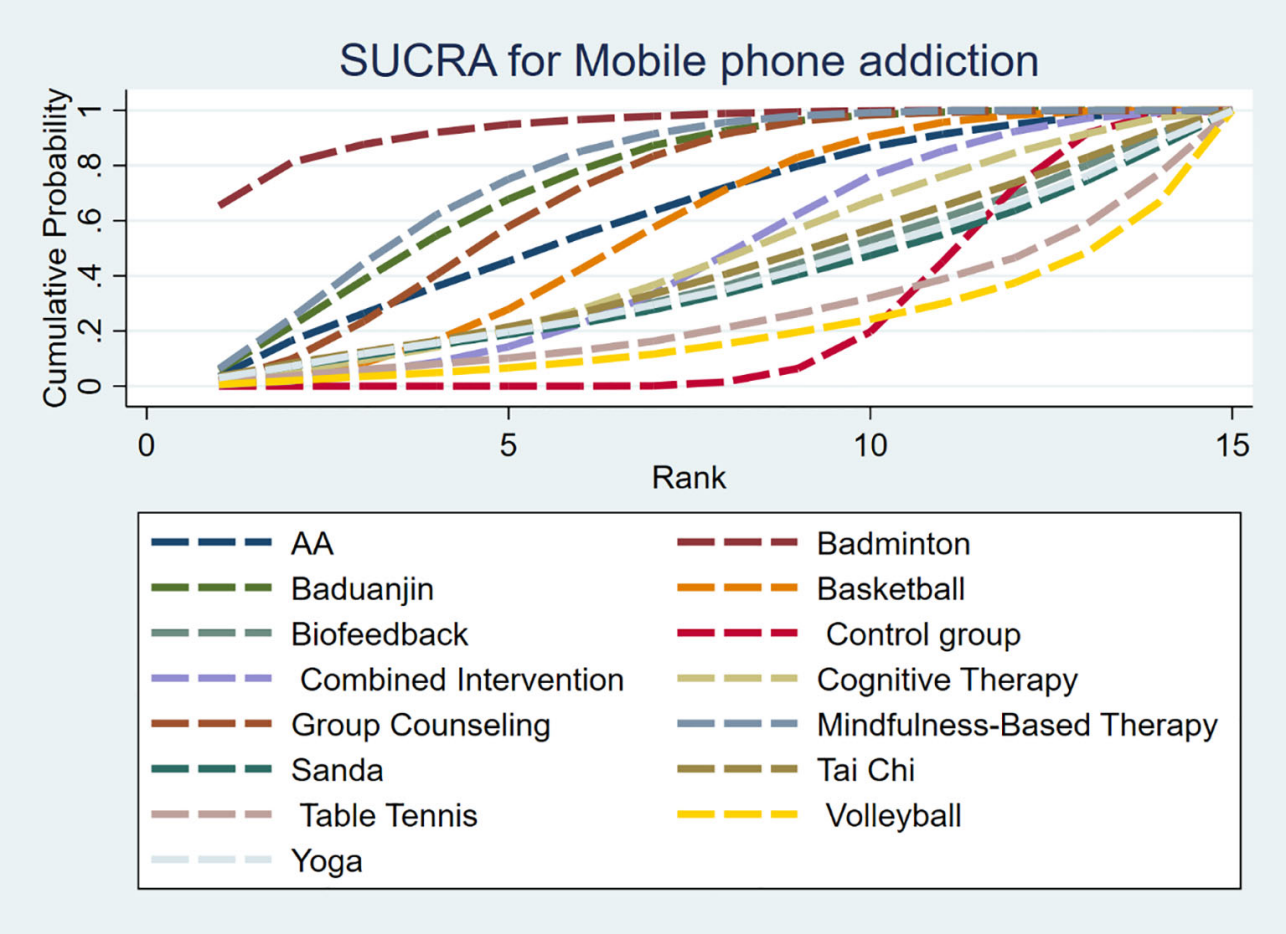
Sucra graph of effectiveness among interventions.

**Figure 8:**
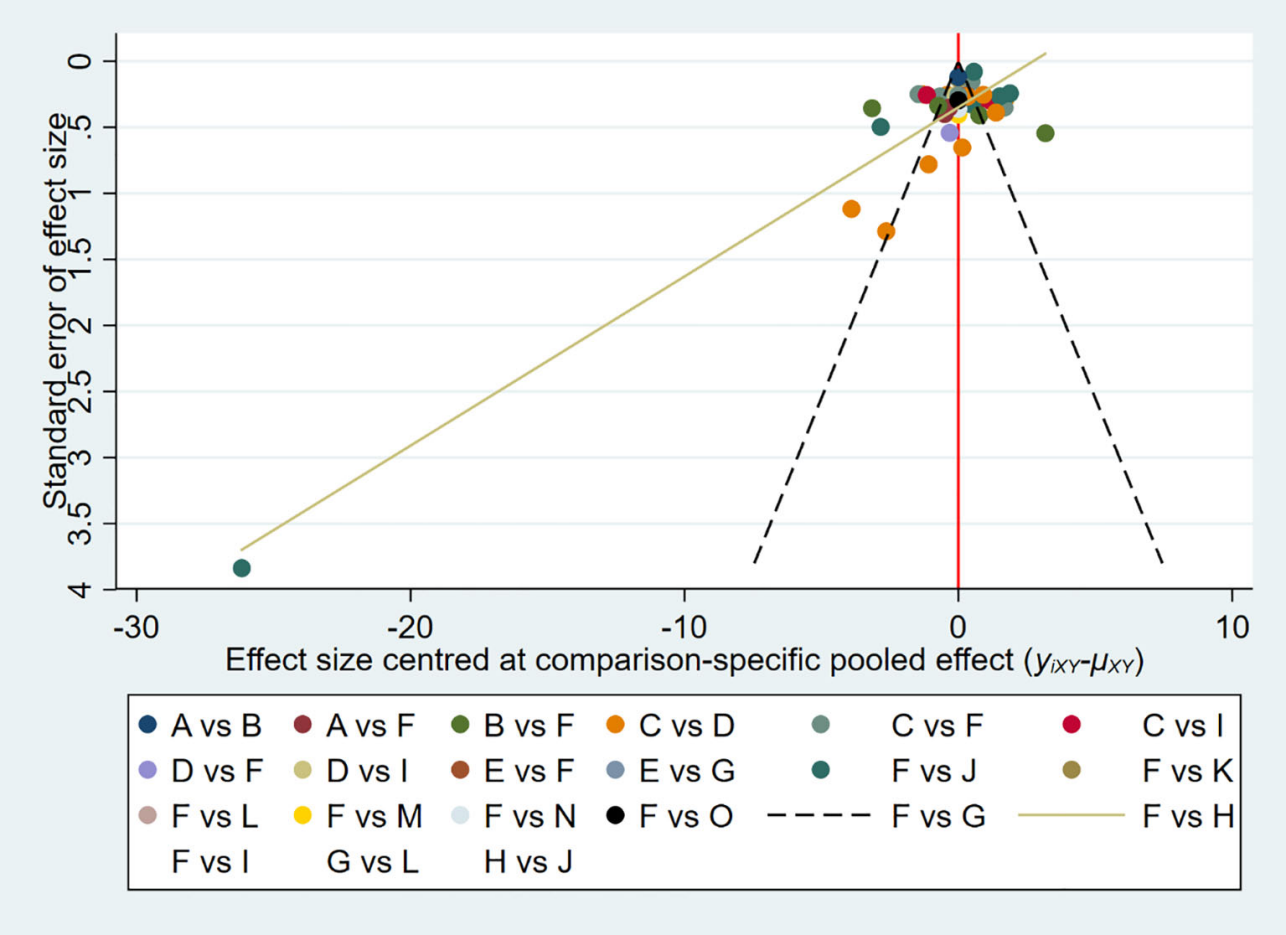
Comparison-adjusted funnel plot of adolescent Internet addiction scores. (A) = Aerobic Aerobics, (B) =Badminton, (C) =Baduanjin, (D) = Basketball, (E) = Biofeedback, (F) =Control group (CG), (G) = Combined Intervention (CI), (H) = Cognitive Therapy (CT), (I) = Group Counseling (GC), (J) = Mindfulness-Based Therapy (MBT), (K) = Sanda, (L) =Tai Chi (TC), (M) =Table tennis (TT), (N) = Volleyball, (O) = Yoga.

The SUCRA probability of each intervention in the network is shown in **Figure 7**. The SUCRA value (**Table 4**) is the probability that each intervention is among the best of those in the network, with larger values representing higher-ranking probabilities. The SUCRA probability of each intervention in the network is shown in **Figure 8**. The SUCRA value (**Table 4**) is the probability that each intervention is among the best of those in the network, with larger values representing higher-ranking probabilities. Badminton (SUCRA=93.8)> MBT(SUCRA=77.3)> Baduanjin (SUCRA=74.3)> GC (SUCRA = 69.6) > Aerobic Aerobics (SUCRA = 62.1) > Basketball (SUCRA = 56.8) > CI (SUCRA = 46.2) > CT (SUCRA = 45.5)> TC (SUCRA = 41.6) >Biofeedback (SUCRA = 39.1) > Yoga (SUCRA = 37.7)> Sanda (SUCRA = 36.3)> TT (SUCRA = 25.7) > CG (SUCRA = 24.0) > Volleyball (SUCRA = 20.0).

**Table 4:** The SUCRA values of the interventions.

Treatment	SUCRA
A= Aerobic Aerobics	62.1
B= Badminton	93.8
C= Baduanjin	74.3
D= Basketball	56.8
E= Biofeedback	39.1
F= Control group (CG)	24.0
G= Combined Intervention (CI)	46.2
H= Cognitive Therapy (CT)	45.5
I= Group Counseling (GC)	69.6
J= Mindfulness-Based Therapy (MBT)	77.3
K= Sanda	36.3
L=Tai Chi (TC)	41.6
M=Table tennis (TT)	25.7
N= Volleyball	20.0
O= Yoga	37.7

#### 3.5.6 Risk of bias across studies

The publication bias was illustrated by funnel plots (**Figure 8**). According to the network meta-analysis, the funnel plot showed slight asymmetry.

## 4 Discussion

Mobile phone addiction seriously endangers the physical and mental health of teenagers or young adults, reduces academic performance and intelligence levels (10, 27). Therefore, it is of great significance to improve mobile phone addiction among teenagers or young adults. This study includes 32 randomized controlled studies. A traditional meta-analysis was conducted on 32 studies to evaluate the effects of the intervention measures and the control group. Furthermore, a network meta-analysis was conducted on the included studies to analyze the direct and indirect comparisons among different intervention measures.

This meta-analysis is based on 32 randomized controlled trials to evaluate the impact of intervention measures on mobile phone addiction among adolescents or young adults. The results showed that compared with the blank control group, all intervention measures (Combined Intervention (CI), Cognitive Therapy (CT), Group Counseling (GC), Mindfulness-Based Therapy (MBT), Exercise intervention) significantly improved adolescent mobile phone addiction. Total amount effect for [SMD = 8.78, 95% CI (10.64, 6.91), p < 0.001), Similar to the results reported by Wu (61) et al. (2023) [SMD= -2.88, 95%CI: (-3.78 to -1.97)] and Pan et al (20) (2023). [SMD=-3.214, 95%CI (-4.293 to -2.135)]. The differences in the subgroup analysis based on Outcome measurement, Intervention duration, and Intervention mode were statistically significant (P < 0.05). However, the differences were not statistically significant in the subgroup analyses of Year of publication, Sample size, and Intervention frequency (p > 0.05). Due to the significant heterogeneity of the analysis results, further analysis and verification are needed.

The conventional meta-analysis confirmed that various interventions demonstrated significantly greater improvement in smartphone addiction compared to control groups. However, this approach is inherently limited to pairwise comparisons, preventing comprehensive evaluation of relative effectiveness across different interventions (20). As an extension of traditional methods, NMA overcomes this limitation by synthesizing both direct and indirect evidence, enabling simultaneous comparison of multiple interventions and identification of the optimal therapeutic strategy (21). Our NMA results show that badminton is superior to other interventions in improving mobile phone addiction among teenagers or young adults. Apart from walking and jogging, badminton is one of the most participated sports among Chinese mass sports enthusiasts, with a participation rate as high as 42.6%. Regular participation in badminton can not only cultivate the reaction ability of both participants but also develop the ability to cooperate with teammates. The interaction during the sport can meet social needs and reduce teenagers’ reliance on obtaining social satisfaction through mobile phones (62). In addition, badminton is characterized by strong competitiveness, high confrontation, and diverse offensive and defensive variations. Badminton participants devote themselves wholeheartedly and pay less attention to their mobile phones (62). Badminton is a moderate-intensity sport. Compared with low-intensity and high-intensity sports, moderate-intensity sports help strike a balance between the benefits of cardiometabolic metabolism and the promotion of pleasant experiences, achieving the promotion of physical and mental health and the replacement and satisfaction of need (63, 64).

Furthermore, our research found that Mindfulness-Based Therapy (MBT) ranked second only to badminton in improving mobile phone addiction among teenagers or young adults. Mind-based intervention is a long-term and delicate training process. During this process, people with an addiction are expected to gradually change their wrong concepts and establish new and objective resistance to inappropriate mobile phone usage behaviors (65). The current mindfulness training process adopts a group mutual assistance model, that is, group members deepen their understanding of positive beliefs and internal viewpoints during the communication process, thereby improving the efficiency of the training (56). During the discussion process, questions need to be answered and mistakes need to be corrected, which enhances the self-control motivation of people with an addiction. Early studies on event-related potential (ERP) have also confirmed that the attention bias caused by the high sensitivity of Internet addiction patients to specific addiction-related cues may be an essential basis for the generation and maintenance of their addictive behaviors (66). The intervention of MBT on mobile phone addiction can be explained from the aspect of attention bias (67). Through MBT, attention bias was trained to a large extent. Attention bias is one of the strong predictors of the recurrence of addictive behaviors.

Although our research found that badminton and MBT have a better effect on improving mobile phone addiction among adolescents or young adults, due to the relatively few randomized controlled trials involving badminton and MBT in this study, the impact of these intervention measures on adolescent Internet addiction still needs to be further explored. Due to the small number of included studies, the results of this study must be preliminarily considered.

### 4.3 Strengths and limitations

It is crucial to identify and explain some advantages and limitations of this study. Our research results confirmed the effectiveness of intervention measures (exercise intervention, MBT, CT, GC) in improving mobile phone addiction among adolescents, and further explored through network element analysis which intervention measure has the best effect on improving mobile phone addiction. First of all, we conducted a comprehensive and systematic search of the published literature to reduce bias and identify potential related studies. Secondly, in this study, the retrieval method was adopted to search seven databases, and the retrieved literature was analyzed. We conducted a strict literature screening. We included 32 studies, and many of the included studies were published in Chinese. Due to the influence of cultural background, their global application is limited. Caution should be exercised when inferring the results. The studies included in this analysis demonstrated unclear allocation concealment and inadequate blinding procedures, methodological limitations that may have introduced performance bias and detection bias, thereby compromising the overall quality of the evidence. We strongly recommend that future studies adhere strictly to standardized reporting of randomization processes and blinding protocols to enhance methodological rigor. Furthermore, our study has high heterogeneity (*I²*= 94%), and high heterogeneity can affect the validity of the meta-analysis results and the reliability of the validity interpretation. Finally, the intervention measures were ranked based on the average score of SUCRA. This does not necessarily mean that the intervention measures with higher rankings are statistically significantly superior to those with lower rankings. Therefore, the research results should be interpreted with caution. Our research confirms that badminton and MBT have a very good effect on improving mobile phone addiction. However, the optimal intervention cycle and frequency remain to be further explored. In future research, attention should be paid to the mobile phone addiction behaviors of different groups of people. The impact of various interventions on the addiction problems of other groups in society should be understood from multiple dimensions, and the health and quality of life of the target population should be improved in a targeted manner. Therefore, in the future, more rigorous, comprehensive and high-quality randomized controlled trials with different cultural backgrounds need to be carried out to provide a reliable theoretical basis for the research update in this field. The mechanism of mobile phone dependence behavior is complex. However, the research has only focused on the impact of psychological intervention and exercise intervention on mobile phone addiction. In the future, the physiological mechanism should be further explored from the perspectives of physiology and imaging.

## 5 Conclusion

According to our research results, all intervention measures have a significant impact on improving Internet addiction. Based on the results of NMA, badminton and MBT may be the best intervention measures. Mobile phone addiction has brought many adverse effects on the physical and mental health of teenagers. In the future, schools should promptly screen for mobile phone addiction among teenagers or young adults, adopt a combination of physical intervention and psychological strategies, and form a comprehensive and systematic intervention system to prevent and reduce the risk of mobile phone addiction among teenagers. However, our research is also subject to some limitations, such as a majority of studies in China, high heterogeneity, and unclear optimal intervention duration/frequency. Therefore, future research should focus on conducting multi-center, cross-cultural randomized controlled trials to further verify the dose-effect relationship of the optimal intervention plan under different cultural backgrounds.

## Data Availability

The original contributions presented in the study are included in the article/**Supplementary Material**. Further inquiries can be directed to the corresponding authors.
