# Supplementary material for: The influence of different intervention measures on improving mobile phone addiction among teenagers or young adults: a systematic review and network meta-analysis
Source: Front Psychiatry. 2025 Sep 17;16:1629251. doi: 10.3389/fpsyt.2025.1629251 (PMC12484140; doi:10.3389/fpsyt.2025.1629251)
Supplement: Supplementary file 1 [file DataSheet1.zip › Supplementary materials/Appendix 1-Search strategy.DOCX]

**Pubmed:**

| Number | Search terms | Results |
| --- | --- | --- |
| **#1** | (((((((((((((Technology Addictions[Title/Abstract]) OR (Mobile Phone Addiction[Title/Abstract])) OR (Addiction, Mobile Phone[Title/Abstract])) OR (Addictions, Mobile Phone[Title/Abstract])) OR (Mobile Phone Addictions[Title/Abstract])) OR (Phone Addiction, Mobile[Title/Abstract])) OR (Cell Phone Addiction[Title/Abstract])) OR (Addiction, Cell Phone[Title/Abstract])) OR (Cell Phone Addictions[Title/Abstract])) OR (Video Game Addiction[Title/Abstract])) OR (Addictions, Video Game[Title/Abstract])) OR (Addiction, Video Game[Title/Abstract])) OR (Game Addiction, Video[Title/Abstract])) OR (Video Game Addictions[Title/Abstract]) | 1311 |
| **#2** | (((((((((((((((((((((((((((((((((((((((((((((((((((((((((((((((((((Aerobic Aerobics[Title/Abstract]) OR (Exercises[Title/Abstract])) OR (Exercise, Physical[Title/Abstract])) OR (Exercises, Physical[Title/Abstract])) OR (Physical Exercise[Title/Abstract])) OR (Physical Exercises[Title/Abstract])) OR (Exercise, Aerobic[Title/Abstract])) OR (Aerobic Exercise[Title/Abstract])) OR (Aerobic Exercises[Title/Abstract])) OR (Exercises, Aerobic[Title/Abstract])) OR (Exercise, Isometric[Title/Abstract])) OR (Exercises, Isometric[Title/Abstract])) OR (Isometric Exercises[Title/Abstract])) OR (Isometric Exercise[Title/Abstract])) OR (Acute Exercise[Title/Abstract])) OR (Acute Exercises[Title/Abstract])) OR (Exercise, Acute[Title/Abstract])) OR (Exercises, Acute[Title/Abstract])) OR (Exercise Training[Title/Abstract])) OR (Exercise Trainings[Title/Abstract])) OR (Training, Exercise[Title/Abstract])) OR (Trainings, Exercise[Title/Abstract])) OR (Physical Activity[Title/Abstract])) OR (Activities, Physical[Title/Abstract])) OR (Activity, Physical[Title/Abstract])) OR (Physical Activities[Title/Abstract])) OR (Racquet Sport[Title/Abstract])) OR (Sport, Racquet[Title/Abstract])) OR (Sports, Racquet[Title/Abstract])) OR (Racket Sports[Title/Abstract])) OR (Racket Sport[Title/Abstract])) OR (Sport, Racket[Title/Abstract])) OR (Sports, Racket[Title/Abstract])) OR (Badminton[Title/Abstract])) OR (Racquetball[Title/Abstract])) OR (Racket Ball[Title/Abstract])) OR (Ball, Racket[Title/Abstract])) OR (Racketball[Title/Abstract])) OR (Squash (Sport[Title/Abstract]))) OR (Squashs (Sport[Title/Abstract]))) OR (Baduanjin[Title/Abstract])) OR (Basketball[Title/Abstract])) OR (Basketballs[Title/Abstract])) OR (Netball[Title/Abstract])) OR (Netballs[Title/Abstract])) OR (Biofeedback[Title/Abstract])) OR (Tai Chi[Title/Abstract])) OR (Tai-ji[Title/Abstract])) OR (Tai Chi[Title/Abstract])) OR (Chi, Tai[Title/Abstract])) OR (Tai Chi Chuan[Title/Abstract])) OR (Taiji[Title/Abstract])) OR (Taijiquan[Title/Abstract])) OR (T'ai Chi[Title/Abstract])) OR (Tai Ji Quan[Title/Abstract])) OR (Ji Quan, Tai[Title/Abstract])) OR (Quan, Tai Ji[Title/Abstract])) OR (Table tennis[Title/Abstract])) OR (Jump rope[Title/Abstract])) OR (Mindfulness-Based Therapy[Title/Abstract])) OR (Cognitive Therapy, Mindfulness-Based[Title/Abstract])) OR (Mindfulness-Based Cognitive Therapies[Title/Abstract])) OR (Mindfulness Based Cognitive Therapy[Title/Abstract])) OR (Therapy, Mindfulness-Based Cognitive[Title/Abstract])) OR (Cognitive Therapy[Title/Abstract])) OR (Sanda[Title/Abstract])) OR (Volleyball[Title/Abstract])) OR (Yoga[Title/Abstract]) | 379,164 |
| **#3** | #1 AND #2 | 105 |
| **#4** | ((Randomized Controlled Trial[MeSH Terms]) OR (RCT[MeSH Terms])) OR (Randomized[Title/Abstract]) | 867,564 |
| **#5** | #3 AND #4 | 17 |
