# Supplementary material for: The influence of different intervention measures on improving mobile phone addiction among teenagers or young adults: a systematic review and network meta-analysis
Source: Front Psychiatry. 2025 Sep 17;16:1629251. doi: 10.3389/fpsyt.2025.1629251 (PMC12484140; doi:10.3389/fpsyt.2025.1629251)
Supplement: Supplementary file 1 [file DataSheet1.zip › Supplementary materials/Appendix 2-Forest plot.docx]

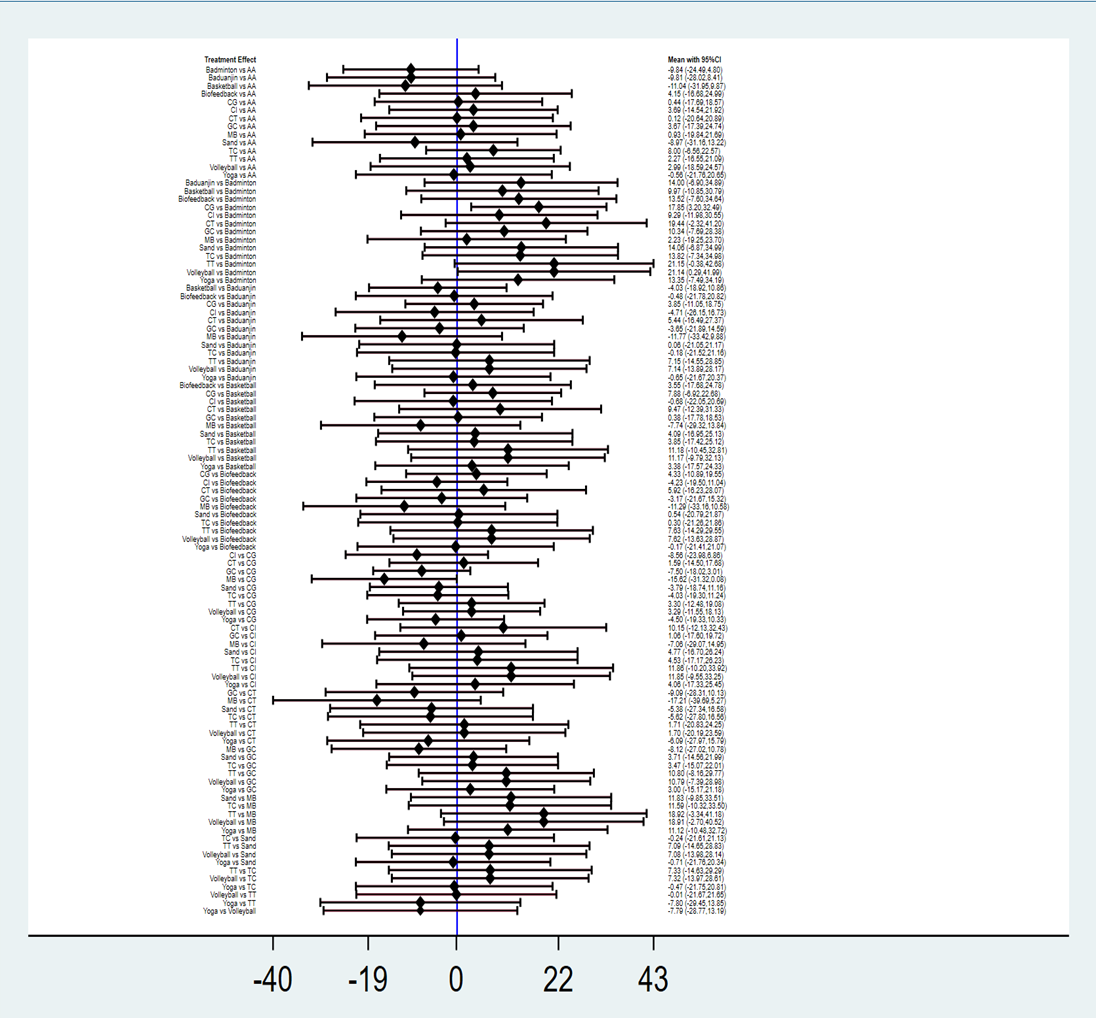


Supplementary figure. Forest plot

***Note:*** Aerobic Aerobics (AA), Tai Chi (TC), Table tennis (TT), Jump rope (JR), Control group (CG), Combined Intervention (CI), Mindfulness-Based Therapy (MBT), Cognitive Therapy (CT), Group Counseling (GC).
